# Sensitivity to numerosity is not a unique visuospatial psychophysical predictor of mathematical ability^☆^

**DOI:** 10.1016/j.visres.2013.06.006

**Published:** 2013-08-30

**Authors:** Marc S. Tibber, Gemma S.L. Manasseh, Richard C. Clarke, Galina Gagin, Sonja N. Swanbeck, Brian Butterworth, R. Beau Lotto, Steven C. Dakin

**Affiliations:** a UCL Institute of Ophthalmology, University College London, Bath Street, London EC1V 9EL, UK; b Neuroscience Program, Wellesley College, Wellesley 02481, USA; c Institute of Cognitive Neuroscience, Dept. Psychology, University College London, London WC1N 3AR, UK; d IRCCS Ospedale San Camillo, Venice, Italy; e School of Psychological Sciences, University of Melbourne, Australia; f NIHR Biomedical Research Centre at Moorfields Eye Hospital NHS Foundation Trust and UCL Institute of Ophthalmology, UK

**Keywords:** Number, Density, Size, Orientation, Mathematics, Spatial vision, IPS, intraparietal sulcus

## Abstract

Sensitivity to visual numerosity has previously been shown to predict human mathematical performance. However, it is not clear whether it is discrimination of numerosity *per se* that is predictive of mathematical ability, or whether the association is driven by more general task demands. To test this notion we had over 300 participants (ranging in age from 6 to 73 years) perform a symbolic mathematics test and 4 different visuospatial matching tasks. The visual tasks involved matching 2 clusters of Gabor elements for their numerosity, density, size or orientation by a method of adjustment. Partial correlation and regression analyses showed that sensitivity to visual numerosity, sensitivity to visual orientation and mathematical education level predict a significant proportion of shared as well as unique variance in mathematics scores. These findings suggest that sensitivity to visual numerosity is not a unique visual psychophysical predictor of mathematical ability. Instead, the data are consistent with mathematics representing a multi-factorial process that shares resources with a number of visuospatial tasks.

## Highlights

• Individual differences in numerosity acuity predict mathematical ability.
• We tested 300+ participants to see if this relationship is unique to numerosity.
• Visual numerosity and orientation task performance predicted mathematics scores.
• Performance improved with age, and males significantly outperformed females.
• This highlights links between mathematics and multiple visuospatial abilities.

## 1 Introduction

Newborn babies are sensitive to the number of elements in a visual display: following habituation to a stimulus newborns fixate longer – a measure of attention, and by inference, perceived novelty – when the number of elements present is changed (Antell & Keating, 1983). Further, newborns will preferentially gaze at a visual stimulus that matches an auditory sequence with respect to the numerosity of component visual objects and auditory events, implicating a role for an innate and abstract representation of numerosity that is independent of sensory modality (Izard et al., 2009). As the infant develops, sensitivity to numerosity improves such that by 10 months they are capable of discriminating 8 from 12 objects (Xu & Arriage, 2007; Xu & Spelke, 2000). According to one group of theories, this preverbal sense of numerosity, which developmentally and anthropologically precedes the acquisition of verbal counting (Feigenson, Dehaene, & Spelke, 2004; Pica et al., 2004), represents the foundation upon which subsequently acquired symbolic and mathematical skills are built (Barth et al., 2005; Gilmore, Mccarthy, & Spelke, 2007; Gilmore & Spelke, 2008). However, there is considerable debate as to precisely how this mapping between representations is established (Carey, 2009; Gallistel, 2007, 2011; Gelman & Butterworth, 2005; Le Corre & Carey, 2007).

There are several strands of evidence to support a close association between non-symbolic numerosity, symbolic number and formal mathematics (Dehaene, 1992). When number–words are mastered number-comparisons based on symbolic information (“which is greater: 3 or 7?”) and non-symbolic information (“are there more blue dots or yellow dots?”) activate common brain regions in the intraparietal sulcus (IPS; Eger et al., 2003; Piazza et al., 2007; Pinel et al., 2004; Venkatraman, Ansari, & Chee, 2005). Participants also show near-identical response patterns for symbolic and non-symbolic number comparison tasks: responses are less precise and slower for the comparison of large numbers (the size effect) and for numbers that are relatively similar in magnitude (the distance effect) (Buckley & Gillman, 1974; Dehaene, Dehaene-Lambertz, & Cohen, 1998; Moyer & Landauer, 1967).

There is also considerable variation *between* subjects with respect to the precision of numerosity judgements. Further, this variation has been shown to retrospectively predict mathematical performance (Halberda, Mazzocco, & Feigenson, 2008). In a study of 14-year-old children, Halberda and colleagues showed that children who were sensitive to differences in visual numerosity tended to have performed well at mathematics. Similar correlations between performance on visual numerosity and mathematics tasks have since been demonstrated in younger (3–6 year old) children (Gilmore, Mccarthy, & Spelke, 2010; Libertus, Feigenson, & Halberda, 2011) as well as adults (Halberda et al., 2012), although in the latter study mathematical ability was primarily inferred on the basis of self-report. [See Inglis et al. (2011) however, for a failure to find such an association in adults.] A link has also been demonstrated between mathematical learning difficulties (Landerl, Bevan, & Butterworth, 2004) and poor performance on visual numerosity tasks (Mazzocco, Feigenson, & Halberda, 2011; Piazza et al., 2010).

Whilst several of these studies have attempted to control for the contribution of cognitive factors common to all tasks, e.g. visual working memory (Halberda, Mazzocco, & Feigenson, 2008; Mazzocco, Feigenson, & Halberda, 2011), participants have not been tested using comparable methods on tasks that involve judgements along other visuospatial dimensions (e.g. size or density). Consequently, it is not clear if it is the discrimination of numerosity *per se* that predicts mathematical performance, or whether the association is driven by other components of the task, e.g. visuospatial attention. To address this issue we tested the hypothesis that sensitivities on other visuospatial tasks are predictive of mathematical performance. Over 300 participants performed a timed computer-based mathematics test followed by 4 visuospatial matching tasks in which 2 patches of Gabors had to be adjusted to match one another with respect to their numerosity, density, size or orientation. Our findings confirm a robust association between mathematics scores and visuospatial sensitivity, but are inconsistent with this connection being specific to visual numerosity: after accounting for the effects of age and general education level, both orientation and numerosity thresholds (as well as mathematical education level) were predictive of mathematics scores.

## 2 Material and methods

Three hundred and thirty-seven participants were recruited at the Science Museum in London on a voluntary basis. Full datasets were gathered on 311 of these (188 male, 6–71 years of age with a mean and standard deviation of 28 ± 14.85 years). All gave informed voluntary consent in accordance with the Declaration of Helsinki.

During a single 30 min testing session participants completed (in order): (1) a questionnaire cataloguing personal details including age, gender, handedness, maximum general and mathematical education level reached; (2) a timed mathematics test; (3) a series of visuospatial matching tasks. General education level was self-scored as an ordinal response (0 = None, 1 = School, 2 = College, 3 = Undergraduate, 4 = Postgraduate). Mathematics education level was scored similarly (0 = None, 1 = GCSE, 2 = A level, 3 = Undergraduate, 4 = Postgraduate). All tests were administered under the supervision of one of the authors or a trained postgraduate assistant. The testing space was a large, secluded room, which was normally lit. Up to 4 participants could be tested at any one time since 4 separate testing ‘stations’ were available for use.

### 2.1 Mathematics test

Mathematical ability was assessed using a computer-based multiple-choice test adapted from the Mathematics Calculation Subtest (WJ-Rcalc) of the Woodcock–Johnson III Tests of Cognitive Abilities (Woodcock & Johnson, 1989). Participants were presented with a series of problems that increased in difficulty from simple addition and subtraction through to multiplication and division of fractions and negative integers. Up to 25 problems could be undertaken, depending on the participant’s performance (see below). These were divided into 5 levels, each of which consisted of 5 problems. On each trial the problem was presented in numeral format (e.g. “5 + 7”) and participants were required to select the correct answer from 4 possibilities (see Supplementary Table 1). No feedback was given. Although response times were recorded, these were not used in the analysis. Participants were given 30 s to respond to each problem, with an icon in the top left-hand corner of the screen indicating the proportion of time remaining on each trial. Failure to respond in the allotted time or selection of an incorrect response (by mouse click) resulted in an error being recorded. An error on 2 sequential levels, or 2 errors on a single level, resulted in termination of the test. The dependent variable was the total number of correct answers given, with 25 being the maximum possible score.

### 2.2 Visuospatial matching tasks

**Fig. 1:**
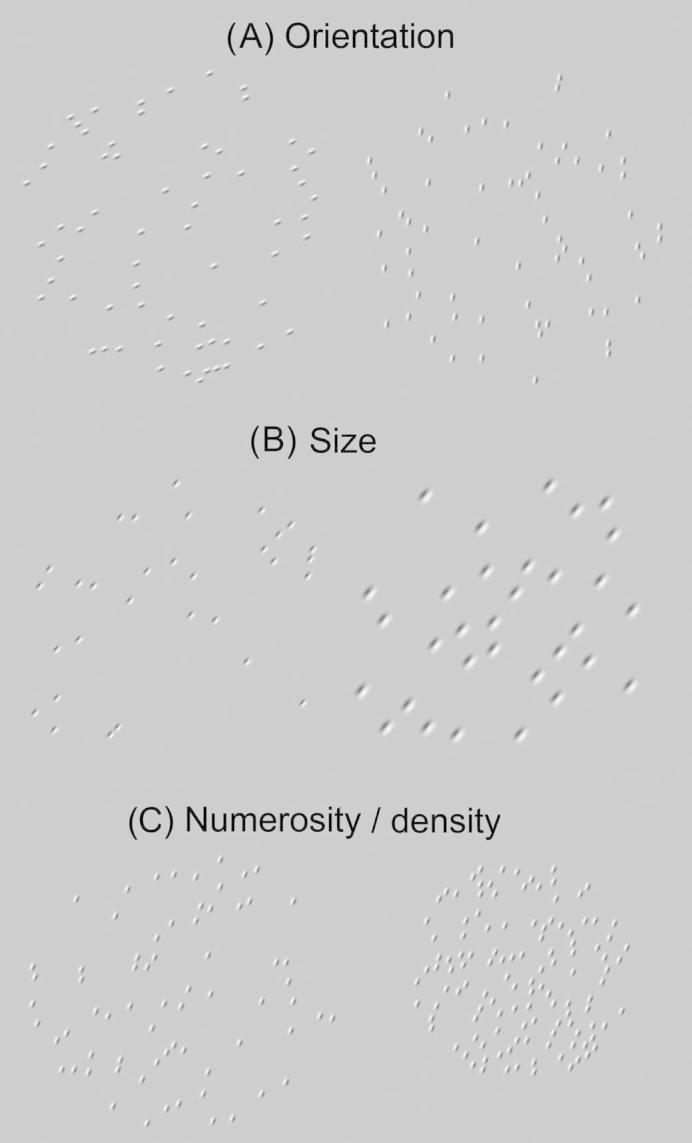
Example stimuli. (A) Orientation, (B) size and (C) numerosity or density tasks.

Two patches of oriented Gabor elements were presented to the left and right of screen centre (Fig. 1). Participants used a mouse to adjust the patch on the right (the test) so that it matched the appearance of the patch on the left (the reference) for a given parameter (orientation, size, numerosity or density). Moving the mouse upwards increased the test parameter value (e.g. the size of elements); moving downwards decreased its value, except for the orientation task, in which upwards and downwards motion mapped onto clockwise and anti-clockwise rotation, respectively. Participants were instructed to click when they could no longer distinguish the two patches with respect to the parameter of interest. To avoid biasing responses through hysteresis (the tendency for a participant’s response to lag behind their perception of a change in stimulus) the initial test patch parameter was chosen at random from the full range of possible test values, thereby randomizing the direction of adjustment. (See Section 2.3 for details of the range of possible test values.) In addition, participants were instructed to hover back and forth about their perceived match point (to ensure a good match) before clicking to indicate a response. This was repeated 4 times per condition and blocked by task in a pseudo-random order. [*Note:* Visual thresholds calculated using a small number of method-of-adjustment trials (5) and a much smaller population sample (*n* = 50) have previously been shown to provide good test/retest reliability and closely concur with parallel measurements made using a 2-alternative forced choice paradigm (Higgins et al., 1988).] At the start of each block the task to be performed was cued and corresponding instructions given.

### 2.3 Stimulus parameters

Stimuli were generated in the Matlab programming environment (MathWorks, Cambridge, MA) using the Psychophysics Toolbox extensions (Brainard, 1997; Pelli, 1997) and were presented on the luminance-calibrated LCD display of an iMac computer at a spatial and temporal resolution of 1920 × 1080 and 60 Hz respectively. Reference and target patches were comprised of a variable number of odd-symmetric-phase, non-overlapping, maximum-contrast Gabor elements presented on a grey background. For the reference patch, the location of each Gabor was updated every second from a random sample of coordinates to prevent participants serial-counting. The position of the elements in the target stimulus did not change, except when the mouse was moved; this led to a re-sampling of element locations.

For tasks 1 and 2 (orientation and size) reference and target patches had a diameter of 10 deg at a viewing distance of 70 cm. For tasks 3 and 4 (numerosity and density) the reference and test had diameters of 10 deg and 7 deg respectively (half an octave difference), thereby decoupling number and density cues (Tibber, Greenwood, & Dakin, 2012). The standard deviation of the Gaussian envelope of the Gabors was fixed at 4.5 arcmins along its longer axis and 3.2 arcmins along the orthogonal axis, except in the size-matching tasks, in which the envelope size varied. The sinusoidal carrier grating typically had a spatial frequency of 5 cycles per degree, but scaled with envelope size in the size task. Gabors were semi-randomly oriented within a range that excluded all angles within 15° of cardinal axes (horizontal/vertical), thereby avoiding the pooling of data from cardinal and oblique axes. The reference and target patch consistently contained 64 elements, except in the size matching condition, in which there were 32 to prevent range restrictions when larger elements were introduced, and the numerosity/density tasks, in which numerosity varied.

The baseline reference level for the parameter of interest in any task (e.g. numerosity in the numerosity matching task) was defined by the standard element described above [*n* = 64, *σ* = 4.5 arcmins, randomly orientated (with constraints)]. However, this baseline was randomly jittered from trial to trial by 0.125 octaves in order to encourage participants to attend to the reference patch rather than rely on an internal standard (Morgan, Watamaniuk, & Mckee, 2000). With respect to the possible range of values that the test stimulus could be moved through: for the orientation task the test orientation ranged between 0° and 180°; for numerosity and density tasks the test numerosity ranged between 2 and 288 elements; for the size task the Gaussian envelope of the Gabors ranged between 0.7 and 12.7 arcmins along the longer axis.

### 2.4 Data analysis

For each task the standard deviation of the perceptual match points (one per trial) provided an estimate of the reliability of the participant’s responses [threshold or Weber fraction (*w*)]. This represents an estimate of the noise in an individual’s underlying numerosity representation. It is thus formally equivalent to *w* as estimated by fitting a single-parameter model to behavioural data describing the change in percent correct as a function of increasing numerosity ratio, as used, for example, by Halberda, Mazzocco, and Feigenson (2008). Lower values indicate better performance. All psychophysical thresholds and age data were log transformed, as this reduced the skewness and kurtosis of their underlying distributions. These were converted into *Z*-scores so that psychophysical data could be filtered identically: values >3 *Z*-scores from the mean were excluded from analyses. This led to the removal of 4.5% of the data. Analyses were carried out using SPSS statistical analysis software (version 18.0; SPSS Inc., Chicago, IL).

## 3 Results

### 3.1 Visuospatial task performance

**Fig. 2:**
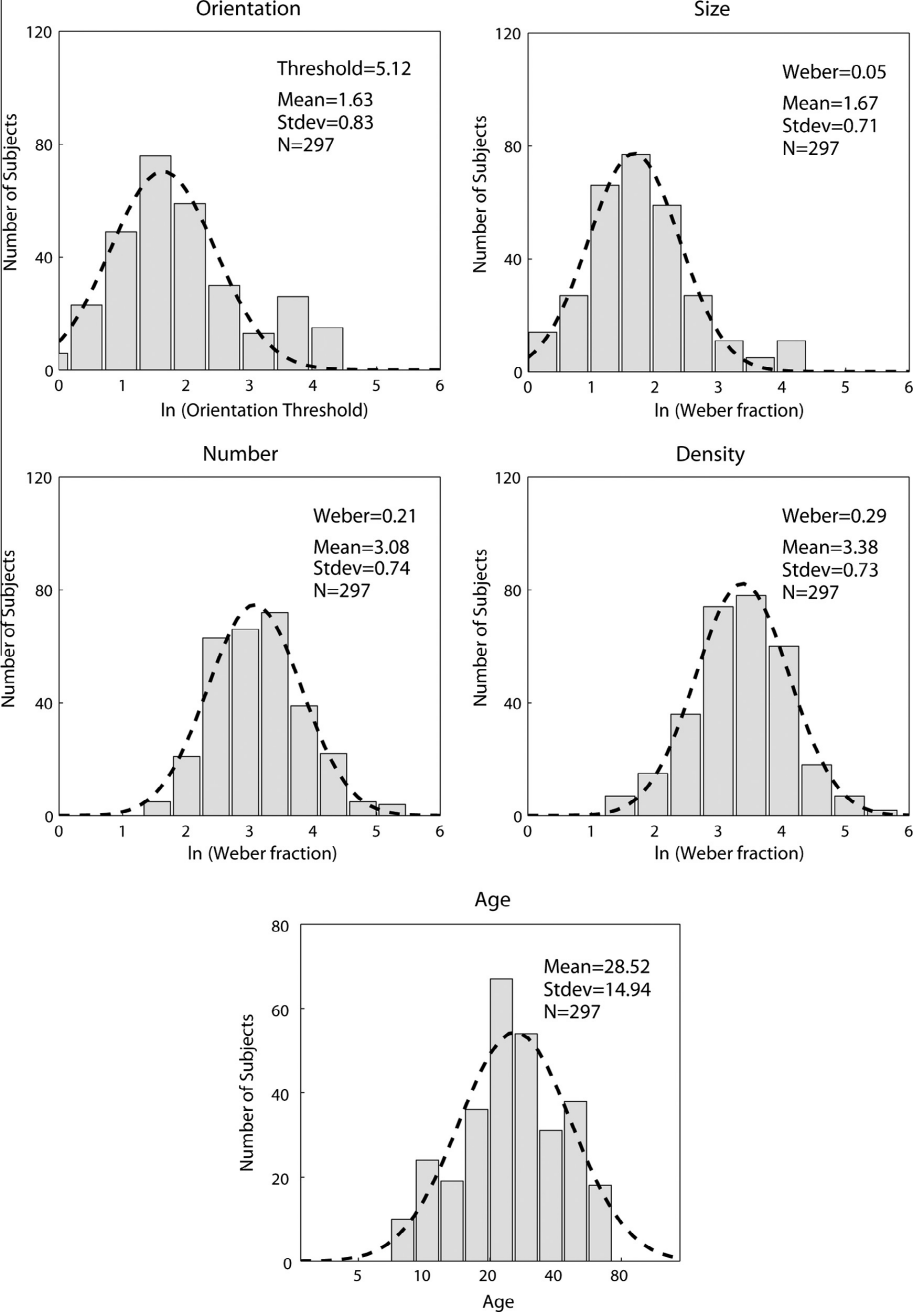
The distribution of log transformed thresholds/Weber fractions and ages. Dashed lines show best-fitting Gaussian functions.

In Fig. 2 log transformed Weber fractions and thresholds (orientation task) are presented for the 4 visuospatial tasks along with the distribution of participants’ ages. Estimates of group means were derived by taking the parameter μ of a Gaussian distribution fit to the log transformed data: orientation (5.12°), size (0.05), number (0.21) and density (0.29). These values are broadly consistent with the existing literature. For example, whilst we report a group mean Weber fraction of 0.21 for numerosity judgements, previous reports in adults (obtained using a range of experimental paradigms and stimuli) typically range from 0.1 to 0.28 (Burr & Ross, 2008; Dakin et al., 2011; Halberda, Mazzocco, & Feigenson, 2008; Piazza et al., 2010; Pica et al., 2004; Ross & Burr, 2010; Tibber, Greenwood, & Dakin, 2012). Thus, we can assume that the method of adjustment employed here provides a reasonable estimate of the population’s performance.

### 3.2 The effects of gender and age on performance

**Fig. 3:**
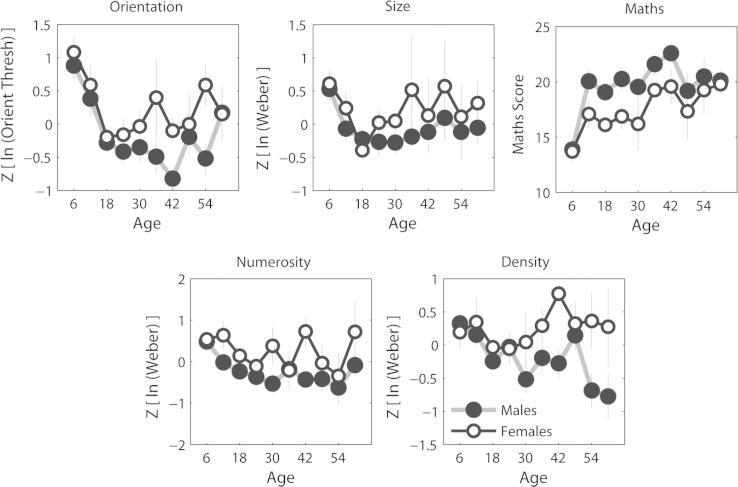
Effects of age and gender on visuospatial and mathematics tasks. Higher (positive) scores represent poorer performance for all tasks except mathematics. For the purposes of graphical presentation and analyses, group data were split by gender and age. See text for further details.

In Fig. 3 task performance is plotted as a function of age for both male and female participants. Visuospatial Weber fractions/thresholds were analyzed in a repeated-measures analysis of variance (ANOVA), with 2 between-subjects factors (gender, at 2 levels, and age, at 10 levels) and one within-subjects factor (task, at 4 levels). The width of age bins selected and the number of participants per bin (in brackets) were as follows: 6–11 (*n* = 37), 13–17 (*n* = 33), 18–23 (*n* = 86), 24–29 (*n* = 46), 30–35 (*n* = 28), 36–41 (*n* = 19), 42–47 (*n* = 20), 48–53 (*n* = 16), 54–59 (*n* = 12), ⩾60 (*n* = 14). All bins were of equal width, with the exception of the last: this captured *all* participants over 60 years of age. This was deemed appropriate as to subdivide this group further would have resulted in extremely small sample sizes. Analyses undertaken revealed no main effect of task (*F*_(3,831)_ = 0.29, *P* = 0.83), but a significant main effect of age group (*F*_(9,277)_ = 6.5, *P* = 2.15 × 10^−8^) and gender (*F*_(1,277)_ = 24.38, *P* = 1.37 × 10^−6^), with no significant interactions. An ANOVA for the mathematics scores revealed an identical pattern with main effects of age (*F*_(9,277)_ = 5.59, *P* = 4.27 × 10^−7^) and gender (*F*_(1,277)_ = 11.33, *P* = 8.69 × 10^−4^), but no interactions. Further tests revealed that these findings reflected superior performance amongst the male participants and an improvement, i.e. lower thresholds and higher mathematics scores, with age (see Supplementary Table 2).

### 3.3 Partial correlations with mathematics scores

**Fig. 4:**
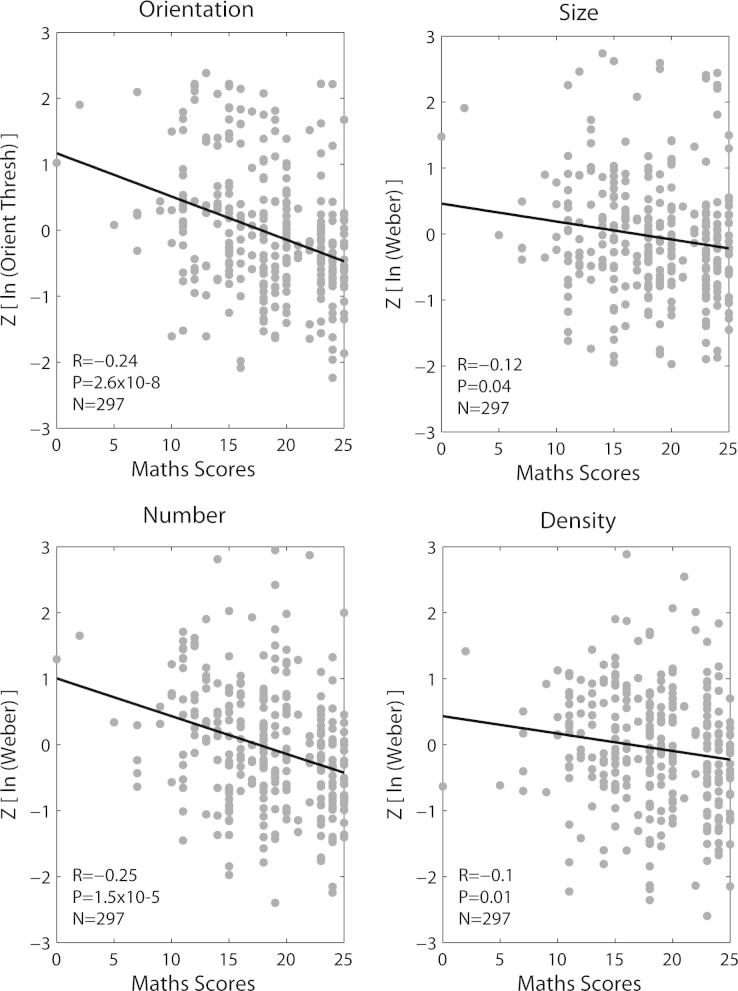
Scatterplot of mathematics scores against visuospatial sensitivity. Correlations reported (details inset) reflect partial correlations with the effects of age held constant. *R* = Pearson’s correlation coefficient; *P* = significance value. Significance values presented are uncorrected for multiple comparisons. See Supplementary Table 2 also.

To determine whether visuospatial task Weber fractions/thresholds correlated with mathematics scores (Fig. 4) we undertook partial correlations between these variables whilst controlling for the effects of age. This was deemed appropriate since age itself correlated highly with performance on several tasks (see Supplementary Table 2). Resulting correlation coefficients were all negative: participants who performed well on the symbolic mathematics test tended to have higher Weber fractions/thresholds for orientation (*R* = −0.24, *P* = 2.6 × 10^−5^), size (*R* = −0.12, *P* = 0.04), numerosity (*R* = −0.25, *P* = 1.5 × 10^−5^) and density (*R* = −0.1, *P* = 0.08) judgements. However, following *Bonferroni* correction for multiple comparisons (28 in total; single-tailed tests) the effects were only significant for orientation and numerosity.

To determine whether this association between mathematical ability and visuospatial sensitivity was restricted to early development, we split the data in two by age to capture adult and pre-adult subpopulations (Group 1 ⩽18 years; Group 2 >18 years) and re-calculated partial correlations for each subgroup. Once again, the effects of age were held constant since age still varied considerably within these two groups. For both orientation and numerosity sensitivity, the correlation with mathematics scores was highly significant in the adult group (*R* = −0.26, *P* = 1.1 × 10^−4^; *R* = −0.26, *P* = 1.2 × 10^−4^; *N* = 216), confirming that the relationship between numerosity sensitivity and mathematics is not restricted to childhood (Halberda et al., 2012). In contrast, in the pre-adult population the effect only approached significance (*R* = −0.2, *P* = 0.08; *R* = −0.21, *P* = 0.06). This most probably reflected a smaller sample size (*N* = 81).

### 3.4 Multiple regression

Having demonstrated a series of robust correlations between measures, we wanted to determine whether orientation and numerosity thresholds would predict a *unique* proportion of variance in mathematics scores once built into the same model. It was not clear that this would be the case, since orientation and numerosity thresholds were highly significantly correlated with one another (Supplementary Table 2: *r* = 0.23, *P* = 7 × 10^−5^). A multiple linear regression analysis was undertaken with mathematics scores as the outcome variable. In model 1 age, orientation thresholds and numerosity Weber fractions were included as predictor variables (Table 1). The resulting model was highly significant (*F*_(3,293)_ = 24.31, *P* < 1 × 10^−6^) and accounted for 20% of the variance in the outcome variable (*R*^2^ = 0.2). This could be broken down further into shared variance (8.55%) and unique variance (11.45%), with contributions from each of the 3 predictor variables. Each of these individually explained a significant proportion of unique variance in the model [age (4.75%), orientation (3.2%) and numerosity (3.5%)].

In Model 2 we tested the possibility that participants’ education history might be mediating some of the effects described (Table 1). Consequently, we re-ran the multiple regression analysis, this time including age, orientation sensitivity, numerosity sensitivity, education level and mathematics education level as predictor variables. [See Norris et al. (2006) on the use of linear regression models with ordinal data.] Once again, the resulting model was highly significant (*F*_(5,291)_ = 18.26, *P* < 1 × 10^−6^) and accounted for 24% of the variance in the outcome variable (*R*^2^ = 0.24). This could be broken down into shared variance (14.5%) and unique variance (9.5%). A significant proportion of unique variance was explained by age (1.7%), orientation (1.7%), numerosity (2.5%) and mathematics education (3.6%) variables. In contrast, general education level was not a significant predictor of mathematics scores once the effects of other factors were controlled for. Size and density thresholds were not included in the regression analyses as they did not correlate with mathematics scores. However, their inclusion in model 2 does not affect the pattern of findings reported (data not presented).

Since mathematical education emerged as a significant predictor of mathematical performance, we wondered whether this variable would similarly predict visual numerosity sensitivity. Partial correlation of mathematical education levels against visual numerosity thresholds (controlling for the effects of age) highlighted a highly significant association (*r* = −0.2, *P* = 5.4 × 10^−4^; Supplementary Table 2). Consequently, we performed a multiple regression analysis with visual numerosity thresholds as the outcome variable (Table 2) and mathematical education level, general education level and age as predictors. The resulting model was highly significant (*F*_(3,293)_ = 8.87, *P* = 1.2 × 10^−5^) and accounted for 8% of the variance in the outcome variable (*R*^2^ = 0.08). However, mathematical education level was the only significant predictor of numerosity thresholds once the effects of other variables were controlled for, explaining 2% of unique variance.

## 4 Discussion

Our results confirm the findings of Halberda and colleagues in demonstrating a significant correlation between mathematical ability and the discrimination of visual numerosity. In addition, our data show that this relationship persists in adulthood (Halberda et al., 2012; Inglis et al., 2011) and holds true for higher numerosities (32–64 elements as opposed to 5–16 in the original study), i.e. well beyond the subitizing range. Further, the strength of the correlation we report (*r* = 0.25 in the pooled data) is similar to that reported previously: with the exception of one study by Halberda and colleagues, which reported an *r* value of 0.5/0.57 (Halberda, Mazzocco, & Feigenson, 2008), *r* values typically fall in a range between 0.2 and 0.4 (Gilmore, Mccarthy, & Spelke, 2010, *r* = 0.38; Libertus, Feigenson, & Halberda, 2011, *r* = 0.26; Halberda et al., 2012, *r* = 0.23). Thus, numerosity sensitivity represents a significant and reliable predictor of mathematics scores. However, orientation sensitivity *also* emerged as a significant predictor of mathematical performance, so that the relationship between mathematics and visuospatial sensitivity is not unique to numerosity.

General factors that potentially link visuospatial sensitivity and symbolic mathematics, and hence may account for shared variance captured in our regression model, include visuospatial working memory and general intelligence (Alloway & Alloway, 2010; Alloway & Passolunghi, 2011; Berry et al., 2010; Dumontheil & Klingberg, 2012; Melnick & Tadin, 2011; Passolunghi, Mammarella, & Altoè, 2008; Raghubar, Barnes, & Hecht, 2010; Rutherford & Troje, 2012). In the original study of 14 year-old children by Halberda, Mazzocco, and Feigenson (2008), general intelligence emerged as a significant predictor of visual numerosity sensitivity once symbolic mathematics and lexical retrieval were controlled for (Table 2 in Halberda, Mazzocco, & Feigenson (2008)), and visual working memory approached significance (*P*s < 0.1, Table 3 in Halberda, Mazzocco, and Feigenson (2008)). Whilst we did not test or control for these factors *independently*, all visuospatial tasks tested were closely matched in terms of procedure and stimulus structure. Nonetheless, any differences in the demands of each task on these factors may have led to variation in the strength of cross-correlations as well as the predictive power of individual measures.

Another potential link between visuospatial sensitivity and mathematics is the need to encode and compare relative magnitudes (Bueti & Walsh, 2009; Walsh, 2003). Whilst several theories of human mathematical development claim a pre-existing numerosity processing network is sequestered for the purposes of symbolic mathematics (Dehaene, Dehaene-Lambertz, & Cohen, 1998), it is not yet clear whether this mechanism is specific to numerosity, or instead, if its location in the parietal cortex reflects a more general association with spatial and magnitude processing (Pinel et al., 2004). There is growing evidence that regions within the IPS encode and compare relative magnitudes independently of perceptual modality or stimulus dimension, i.e. rather than *numerosity* exclusively (Cappelletti, Muggleton, & Walsh, 2009; Eger et al., 2003; Piazza et al., 2006, 2007; Pinel et al., 2004; Venkatraman, Ansari, & Chee, 2005). For example, transcranial magnetic stimulation (TMS) of the IPS impairs judgements of relative size as well as relative numerosity, but critically, does not affect performance on a task that involves the processing of symbolic numerals without a comparison of quantity (Cappelletti, Muggleton, & Walsh, 2009). In addition, inter-individual differences in activity within this region during a visual working memory task have been shown to predict mathematical performance 2 years subsequently (Dumontheil & Klingberg, 2012).

Although an association between visuospatial and mathematical processing is consistent with the literature discussed above, it is nonetheless unclear why the association we report was limited to a subset of visuospatial tasks. Thus, why did orientation and numerosity thresholds predict mathematics scores, whilst those for density and size did not? Further, what is the source of the unique variance in mathematics scores that could be explained by individual visuospatial tasks? Particularly puzzling is the lack of an association between mathematical performance and density thresholds since we have previously demonstrated an intimate link between numerosity and density judgements (Tibber, Greenwood, & Dakin, 2012) and provided a relatively simple model of their completion based on a common filtering stage (Dakin et al., 2011). [See Stoianov and Zorzi (2012) also for details of a related network model of numerosity perception.] Hence, if numerosity thresholds were predictive of mathematics scores we would strongly expect density thresholds to be so also. However, the fact that thresholds reported were considerably higher for density than they were for numerosity (0.29 compared to 0.21), a finding that directly contradicts previous results, including our own (Dakin et al., 2011; Ross & Burr, 2010; Tibber, Greenwood, & Dakin, 2012), leads us to speculate that a subset of participants may have used inappropriate or mixed strategies for the density task. Indeed, there was a potential ambiguity with respect to whether participants should match the patches for *absolute* density (i.e. the actual inter-element spacing), or match the patches for *relative* density according to overall patch size (so that the reference and test appeared to be scaled versions of one another). The possibility that density judgements are actually predictive of mathematical performance, but that the effect was diluted by a subset of participants performing suboptimally is also consistent with the finding that density thresholds correlated with mathematical scores prior to *Bonferroni* correction for multiple comparisons (Supplementary Table 2).

Similarly, we can only speculate as to why size thresholds did not predict mathematical performance when other visuospatial tasks, i.e. numerosity and orientation judgements, did. Unlike density however, size thresholds did not even approach significance as a correlate of mathematical performance, suggesting that in some fundamental way size judgements differ from the other 3 visuospatial tasks (Supplementary Table 2). One possibility is that size judgements place a lower load on available (potentially shared) resources, thereby reducing the likelihood of detecting any association, although why this would be the case is not clear. Consistent with this possibility however, Weber fractions for size were much lower than for number or density judgements, 0.05 compared to 0.21 and 0.29, respectively (Fig. 2). Although merely speculative, this possibility lends itself to a clear prediction, namely that size judgements should interfere less with simultaneously executed numerosity judgements than would orientation judgements in a dual-task interference paradigm. An alternative interpretation is that size judgements simply tap into distinct (independent) mechanisms, although the afore-mentioned findings from TMS studies of the IPS suggest otherwise (Cappelletti, Muggleton, & Walsh, 2009).

Halberda, Mazzocco, and Feigenson (2008) provided two possible interpretations of their data: first, that inter-individual differences in an innate mechanism play a causal role in the development of both mathematical and visuospatial abilities; second, that experience in symbolic mathematics leads to learning that is transferred to performance on visuospatial judgements. Our data are largely consistent with the second hypothesis. Thus, we have shown that mathematical education levels significantly predict both mathematics scores *and* sensitivity to visual numerosity, even when other factors are built into the model (i.e. age and general education level). Thus, having a higher level of education in mathematics is associated with high orientation and numerosity sensitivity as well as greater mathematical ability. Since mathematical education does not involve training in orientation and numerosity judgements, it clearly cannot be the case that learning is transferred in the opposite direction: i.e. from visuospatial to symbolic mathematics tasks. One possibility that should be considered however, is that a common system does indeed subserve both mathematical and visuospatial tasks, and that people with a sensitive mechanism are simply more likely to pursue mathematics education to a higher level. Unfortunately the data are unable to establish any direction of causality. However, it is clear that even if training in mathematics *were* shown to transfer to performance on numerosity tasks, other factors must also play a role in the underlying mechanism’s developmental trajectory, since number acuity improves dramatically prior to the child receiving any training in symbolic mathematics (Halberda & Feigenson, 2008; Piazza et al., 2010).

Finally, we also provide evidence that male participants tended to outperform females on all visuospatial and mathematical tasks administered. Nonetheless, this is not necessarily indicative of a biological (genetic) underpinning. Whilst a number of previous studies report male participants outperforming females at mathematics (Fryer & Levitt, 2010; Hyde, Fennema, & Lamon, 1990; Mullis et al., 2000; Rosselli et al., 2009) as well as a subset of visuospatial tasks that require mental transformation of stimuli (Delgado & Prieto, 1996; Lawton & Hatcher, 2005; Loring-Meier & Halpern, 1999; Masters, 1998; Masters & Sanders, 1993; Voyer, Voyer, & Bryden, 1995), the performance gap (where detected) has been shown to widen with age and is likely to reflect differences in cultural expectations and exposure history (Hyde, Fennema, & Lamon, 1990; Lindberg et al., 2010).

## 5 Conclusions

The data presented support the notion of an intimate association between mathematics and visuospatial sensitivity. However, the findings also indicate that this relationship is not unique to visual numerosity, raising the question: what connects these seemingly disparate tasks? Whilst several possibilities have been discussed, from cognitive components such as visuospatial working memory and general intelligence or the notion of a common magnitude comparison system, it seems unlikely that any single factor will capture the data in its entirety. Thus, orientation sensitivity, numerosity sensitivity and mathematical education level all contributed to the prediction of unique as well as shared variance in mathematics scores, suggesting partially overlapping mechanisms. Future studies will therefore need to employ a broader range of closely matched tests and cognitive manipulations if the precise nature of mathematics’ composite processes are to be unravelled.

## Figures and Tables

**Table 1:** Multiple regression – predicting mathematics scores. For each model listed (1 and 2), all predictor variables reported were added simultaneously rather than hierarchically. *P* values in bold denote minimum significance at an alpha level of 0.05.

Model no.	Variable	Beta	*t*	*P*
1	Age	0.23	4.18	**3.9** **×** **10^−5^**
	Orientation	−0.19	−3.4	**1** **×** **10^−3^**
	Numerosity	−0.2	−3.58	**4** **×** **10^−4^**

2	Age	0.17	2.58	**0.01**
	Orientation	−0.15	−2.53	**0.01**
	Numerosity	−0.17	−3.09	**2** **×** **10^−3^**
	Education	−0.08	−1	0.32
	Maths education	0.27	3.72	**2.4** **×** **10^−4^**

**Table 2:** Multiple regression – predicting numerosity sensitivity. For model 2, all predictor variables reported were added simultaneously. *P* values in bold denote minimum significance at an alpha level of 0.05.

Model No.	Variable	Beta	*t*	*P*
1	Maths education	−0.27	−4.89	**2** **×** **10^−6^**
2	Maths education	−0.2	−2.61	**0.01**
	Education	−0.04	−0.48	0.63
	Age	−0.08	−1.23	0.22

## Appendix A Supplementary material

Supplementary Table 1 Questions, answers and distracters used in the symbolic mathematics test. Participants were presented with up to 25 mathematics questions (5 per each of 5 levels) according to performance. Possible answers that the participant could chose from are presented in brackets, with the correct answer presented first. During the experiment the spatial presentation order of possible answers was randomised on each trial.

Supplementary Table 2 Zero-order and partial correlations. Pearson’s correlation coefficients (*R*) and corresponding significance levels (*P*) are shown for correlations between age, mathematics score, general education level (Ed), mathematics education level (MEd) and psychophysical task performance (with age and Weber fractions/thresholds log transformed and converted into *Z*-scores). With the exception of the top row, which reports zero-order correlations between age and other variables, all values presented reflect partial correlations with the effects of age held constant. Education level data are based on self-report and represent ordinal responses to a questionnaire (General education: 0 = None, 1 = School, 2 = College, 3 = Undergraduate, 4 = Postgraduate; Mathematics education: 0 = None, 1 = GCSE, 2 = A level, 3 = Undergraduate, 4 = Postgraduate). *P* values reported have not been corrected for multiple comparisons. ^*^Correlations that are significant at the 5% level minimum (uncorrected); ^**^Correlations that are significant at the 5% level following *Bonferroni* correction for multiple comparisons (28 in total; single-tailed tests). *N* = 297 participants.
