# Supplementary material for: Sensitivity to numerosity is not a unique visuospatial psychophysical predictor of mathematical ability
Source: Vision Res. 2013 Aug 30;89:1–9. doi: 10.1016/j.visres.2013.06.006 (PMC3748346; doi:10.1016/j.visres.2013.06.006)
Supplement: Supplementary Table 1 — Questions, answers and distracters used in the symbolic mathematics test. Participants were presented with up to 25 mathematics questions (5 per each of 5 levels) according to performance. Possible answers that the participant could chose from are presented in brackets, with the correct answer presented first. During the experiment the spatial presentation order of possible answers was randomised on each trial. [file mmc1.doc]

|  | **Q.1** | **Q.2** | **Q.3** | **Q.4** | **Q.5** |
| --- | --- | --- | --- | --- | --- |
| **Level 1** | 6 + 1  [7,1,5,2] | 2 + 4  [6,2,7,8] | 3 - 2  [1,3,2,4] | 5 - 2  [3,1,4,8] | 3 - 1  [2,0,3,6] |
| **Level 2** | 5 – 1  [4,2,5,7] | 9 + 7  [16,12,15,23] | 17 – 9  [8,26,5,11] | 89 – 18  [71,56,107,75] | 5 x 3  [15,45,18,25] |
| **Level 3** | 8 ÷ 2  [4,6,16,2] | 8 x 5  [40,45,85,30] | 13 x 7  [91,37,81,107] | 48 – 19  [29,23,30,22] | 14 x 6  [84,64,72,52] |
| **Level 4** | 2/3 – 1/3  [1/3,1/6,1/2,2/9] | 126 ÷ 42  [3,4,4 1/2,5] | 288 ÷ 48  [6,7,7 1/2,9] | 7/8 – 2/8  [5/8,3/5,1 3/5,1/8] | 3250 / 25  [130,50,80,110] |
| **Level 5** | 2 3/4 + 4 1/8  [6 7/8,6 1/2, 8 1/2,7 1/4] | 1.05 x 0.2  [0.21,2.1,0.3,2.2] | -18 + 12  [-6,6,-30,30] | -6 x 7  [-42,42,-67,1] | 4/7 ÷ ½  [8/7,1 ¼,2/7,1/7] |
