# Supplementary material for: Sensitivity to numerosity is not a unique visuospatial psychophysical predictor of mathematical ability
Source: Vision Res. 2013 Aug 30;89:1–9. doi: 10.1016/j.visres.2013.06.006 (PMC3748346; doi:10.1016/j.visres.2013.06.006)
Supplement: Supplementary Table 2 — Zero-order and partial correlations. Pearson’s correlation coefficients (R) and corresponding significance levels (P) are shown for correlations between age, mathematics score, general education level (Ed), mathematics education level (MEd) and psychophysical task performance (with age and Weber fractions/thresholds log transformed and converted into Z-scores). With the exception of the top row, which reports zero-order correlations between age and other variables, all values presented reflect partial correlations with the effects of age held constant. Education level data are based on self-report and represent ordinal responses to a questionnaire (General education: 0 = None, 1 = School, 2 = College, 3 = Undergraduate, 4 = Postgraduate; Mathematics education: 0 = None, 1 = GCSE, 2 = A level, 3 = Undergraduate, 4 = Postgraduate). P values reported have not been corrected for multiple comparisons. *Correlations that are significant at the 5% level minimum (uncorrected); **Correlations that are significant at the 5% level following Bonferroni correction for multiple comparisons (28 in total; single-tailed tests). N = 297 participants. [file mmc2.doc]

|  |  | **Age** | **Maths** | **Orient** | **Size** | **Num** | **Den** | **Ed** | **Med** |
| --- | --- | --- | --- | --- | --- | --- | --- | --- | --- |
| **Age** | R |  | 0.34 | -0.32 | -0.09 | -0.21 | -0.14 | 0.59 | 0.49 |
|  | P |  | 3.1x10^-9^** | 2.3x10^-8^** | 0.134 | 2x10^-4^** | 0.02* | 9.5x10^-30^** | 2.1x10^-19^** |
| **Maths** | R |  |  | -0.24 | -0.12 | -0.25 | -0.10 | 0.14 | 0.29 |
|  | P |  |  | 2.6x10^-5^** | 0.04* | 1.5x10^-5^** | 0.08 | 0.02* | 3.7x10^-7^** |
| **­­Orient** | R |  |  |  | 0.26 | 0.23 | 0.15 | -0.26 | -0.29 |
|  | P |  |  |  | 6.7x10^-6^** | 7x10^-5^** | 0.01* | 5.8x10^-6^** | 4.9x10^-7^** |
| **Size** | R |  |  |  |  | 0.35 | 0.20 | -0.16 | -0.22 |
|  | P |  |  |  |  | 3.8x10^-10^** | 4.3x10^-4^** | 0.01* | 1.7x10^-4^** |
| **Num** | R |  |  |  |  |  | 0.34 | -0.14 | -0.20 |
|  | P |  |  |  |  |  | 1.2x10^-9^** | 0.02* | 5.4x10^-4^** |
| **Den** | R |  | ­­ |  |  |  |  | -0.08 | -0.14 |
|  | P |  |  |  |  |  |  | 0.19 | 0.02* |
| **Ed** | R |  |  |  |  |  |  |  | 0.57 |
| ­­­­­ | P |  |  |  |  |  |  |  | 1.2x10^-26^** |
| **MEd** | R |  |  |  |  |  |  |  |  |
